# Assessing Competency and Training of Upper Endoscopy in a General Surgery Residency Program

**DOI:** 10.4021/gr520w

**Published:** 2013-10-31

**Authors:** William F. Powers, W. Borden Hooks, S. Nicole Kilbourne, Thomas V. Clancy, William W. Hope

**Affiliations:** a Department of Surgery, South East Area Health Education Center, Department of Surgery, New Hanover Regional Medical Center, Wilmington, North Carolina, USA

**Keywords:** Endoscopy, Education, Curriculum

## Abstract

**Background:**

Guidelines for optimal endoscopic training for surgical residents have not been formally integrated into modern teaching programs. Our purpose was to apply two endoscopic evaluation tools (EE-1 and EE-2) designed to measure surgical resident competency in the performance of esophagogastroduodenoscopy (EGD).

**Methods:**

Prospectively collected data were reviewed from consecutive EGDs in a single institution by a single attending surgeon over 3 years (July 2008 to July 2011). Demographic, procedural, and outcome data were collected. Residents were graded at the completion of each procedure by the attending surgeon using EE-1 and EE-2. Descriptive statistics were calculated, and comparisons based on PGY levels were made using Fisher’s exact and Kruskal-Wallis tests. P < 0.05 was considered significant.

**Results:**

All procedures (N = 50) were performed by residents under the direct attending surgeon supervision. Average patient age was 51 years (range, 31-79 years), 66% were women, and 66% were Caucasian. PGY-3 residents performed 62% of the procedures. Average resident participation was 84% of each procedure. Biopsies were performed in 80% of patients and dilatations in 16%. All EGDs were successfully completed (average time, 13.1 min). EE-1 results demonstrated significantly different grades (P < 0.05) among PGY levels in seven of eight variables. EE-2 grades were significantly different (P < 0.05) among PGY levels in all 10 variables with a general trend of improvement as PGY level increased. There were no mortalities or morbidities.

**Conclusions:**

Residents can perform EGDs safely and expeditiously with appropriate supervision. Methods to assess competency continue to evolve and should remain an area of active research.

## Introduction

Diagnostic endoscopy is an integral part of the general surgeon’s practice. Training in endoscopic technique has been a cornerstone in the general surgery curriculum since 1980 when it became mandatory by the American Board of Surgery. The importance of endoscopic training during residency has been well documented by graduates [1] and program directors [2, 3], since endoscopy is a significant portion of surgical practice following residency completion.

Recently, several issues related to the lack of uniform criteria for competency validation have prompted surgical residency programs to reassess how best to teach and document the effectiveness of their endoscopic training curricula. The Accreditation Council for Graduate Medical Education in a 2009 decision mandated that the minimum number of endoscopic procedures completed during residency be increased to 35 upper (esophagogastroduodenoscopy (EGD)) and 50 lower (colonoscopies) for graduating residents. This requirement was likely influenced by the conclusions of a single prospective study by Wexner et al in 2001 [4]. The change has prompted introspection by many surgical residency programs as they try to develop validated endoscopic training curricula and has refueled the controversy among endoscopic societies who espouse a specialty-based rather than data-based standard for what constitutes the optimal number of procedures prerequisite for competent endoscopic practice. The current interest in developing sensible endoscopic standards and the paucity of data to validate specific numbers-based criteria have prompted programs to study this topic.

The purpose of this study was to evaluate two tools designed to measure general surgical resident competency in the performance of EGD.

## Materials and Methods

This study was approved by the Institutional Review Board at our medical center. Prospectively collected data were reviewed from consecutive EGD procedures from a single surgeon from July 2008 to July 2011. There was no formal endoscopy curriculum or rotation during the time of the study. Upper endoscopies performed without surgical residents were excluded. Demographic information, procedural data, and outcome data were collected. Residents were graded by the attending surgeon using two different endoscopic evaluation tools, EE-1 and EE-2, at the completion of each procedure. EE-1 evaluated knowledge and technical skill with eight criteria: 1) basic knowledge, 2) knowledge of anatomy, 3) ability to intubate the esophagus, stomach, and duodenum, 4) performance of techniques, 5) performance of critical portion of procedure (length of z-line, retroflexion, ability to enter the second portion of duodenum), 6) prevention of complications, 7) time/flow of procedure, and 8) overall performance. All criteria were equally weighted on a scale of 5 for a maximum score of 40.

EE-2 graded 10 different generic skills: 1) handling of scope, 2) handling of controls, 3) force used, 4) hand, eye, and motor coordination, 5) patient discomfort, 6) flow of procedure and specific endoscopic skills, 7) luminal vision, 8) therapeutic procedure, 9) strategy for progression, and 10) identifies end landmarks. The maximum score for the 10 components is 70 [5]. Both tools used a five-point Likert scale (1 = poor and 5 = excellent). Descriptive statistics were calculated, and comparisons were made based on postgraduate year of training using Fisher’s exact test and Kruskal-Wallis test, with a P < 0.05 considered significant.

## Results

All 50 EGD procedures were performed by residents and graded by the attending surgeon. The average age was 51 years (range, 31-79 years). Women comprised 66% of patients and 66% were Caucasian. The procedures were performed by PGY-2 residents in 10% of cases, PGY-3s in 62%, PGY-4s in 8%, and PGY-5s in 20% of cases. The average resident participation was 84% of the procedure. Biopsies were performed in 80% and dilatations in 16%. Barrett’s esophagus was present in 6% of patients, and 4.4% had a positive Campylobacter-like organism test. All procedures were successfully completed with an average procedure time of 13.1 min (range, 4-43 min). There were no mortalities or morbidities.

Residents were assessed using both assessment tools in 46 cases. Among this group of patients, 37 underwent biopsies. Using EE-1, the average resident score in 37 patients undergoing biopsy was 24 of a possible 40. In the nine patients not undergoing biopsy, the average resident score was 24 of a possible 35. Using EE-2, the average resident score in patients undergoing biopsy was 47 of a total 70 and 39 of a total 60 in patients not undergoing a biopsy.

**Figure 1:**
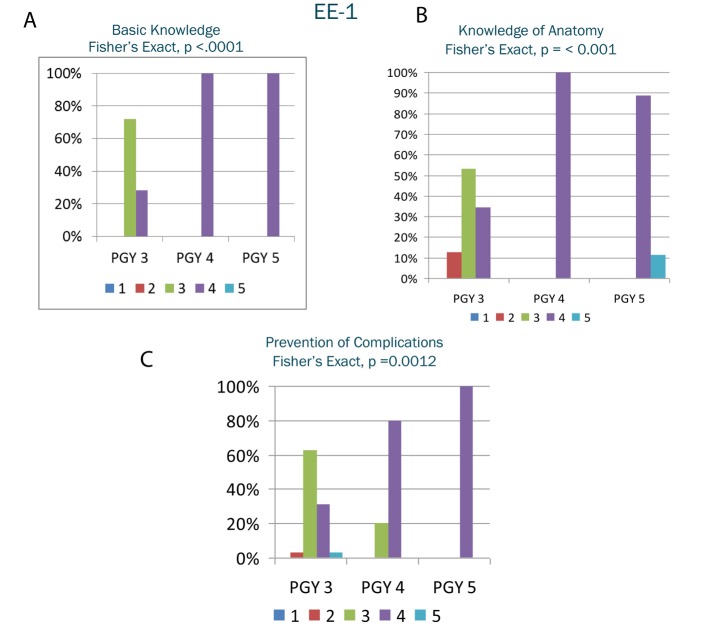
A-C. Selected results from endoscopic evaluation 1. The y-axis represents the percentage of residents in the postgraduate year receiving a particular grade. The x-axis represents the postgraduate year. The shaded columns within the postgraduate year represent the grade received on the Likert scale.

**Figure 2:**
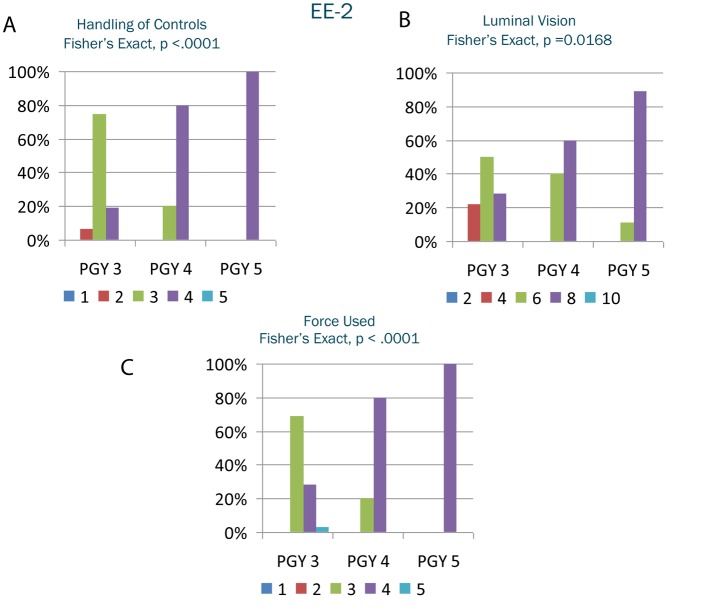
A-C. Selected results from endoscopic evaluation 2. The y-axis represents the percentage of the postgraduate year receiving a particular grade. The x-axis represents the postgraduate year. The shaded columns within the postgraduate year represent the grade received on the Likert scale.

EE-1 results (Fig. 1) demonstrated significantly different grades (P < 0.05) among PGY levels in seven of the eight EE-1 variables: basic knowledge, knowledge of anatomy, performance of techniques, performance of critical portion of procedure, prevention of complications, time and flow of procedure, and overall categories with a general trend of improvement as PGY increased. EE-2 results (Fig. 2) were significantly different (P < 0.05) among PGY levels in all 10 variables with a general trend of improvement as PGY increased. There was not a significant difference between PGY and procedure time (P = 0.1).

## Discussion

Endoscopic procedures comprise a substantial portion of a general surgeon’s practice [6-8]. The need for evidence-based teaching and practice performance measures have been illuminated by public demand for excellence, low cost, and no complication medical care [9]. Concomitantly but not necessarily complementary, resident duty hour restrictions designed to lower iatrogenic morbidity and mortality through fatigue reduction contribute to a reduction in physician exposure to even routine procedures and overall time in training [5, 10]. The era of “see one, do one” has ended as we strive to integrate more objective skills assessment tools into already compressed time regulations.

Skill acquisition is developed over time with patience and practice. Recognition of a problem is facilitated by having experienced similar situations; however, not every skill requires the same time or practice to master for each individual. By separating endoscopic skills into groups of tasks that are appropriate for a given trainee level, a progression of competency in each skill is attained in a measurable fashion [11].

Our study used two evaluation tools to assess resident competency in the performance of EGD [5]. During the completion of our prospective data collection process, Vassiliou et al reported a new validated endoscopic assessment tool, the global assessment of gastrointestinal endoscopic skills (GAGES) [12], which we have incorporated into our assessment methods but do not have sufficient data to report. Our current data suggest that our residents across all PGY levels can safely perform EGDs under appropriate supervision according to the basic skill sets that were measured with the validated scales. The general trend observed was that as PGY level increased, so did performance in the measured metrics on both assessment scales. There were no significant differences in performance scores when a biopsy was not performed using both assessment tools, which may be explained by the procedure being technically less difficult. The finding of no significant difference in overall time based on PGY level can possibly be explained by the short duration of these cases and lack of technical difficulty compared with more complex endoscopic procedures such as colonoscopy.

Our study is limited by its small size and having only one attending to assess residents despite using two separate grading scales. Our residency program had no formal endoscopy rotation or curriculum throughout the time of this study. Residents were taught in the endoscopy suite and allowed to gradually increase the percentage of the case they performed based on skill level while maintaining a safe and appropriate exam as observed by the attending surgeon.

We have recently implemented a formal endoscopy curriculum based on a growing instructive literature and the availability of new technologic and educational tools for ensuring optimal teaching methods. It includes both upper and lower endoscopic procedures, didactic lectures from surgeons and gastroenterologists, video education, and simulation-based training with assistance provided by the surgeons training endoscopic proficiency (STEP) program. We will also incorporate the fundamentals of endoscopic surgery (FES) [13] curriculum from SAGES as it becomes available for residency program implementation. Data will be collected throughout the training process to compare before and after results in an effort to demonstrate that surgical residents can achieve endoscopic competency in a safe and measurable environment. Whether the number of cases will independently predict optimal performance remains to be determined.

In conclusion, residents at the PGY 2-5 levels can safely and competently perform EGD. As PGY levels increase, several of the skills required for EGD performance improve. However, we did not observe this significantly decreasing time required for the procedure. The implementation of a formal endoscopy curriculum with validated assessment tools will help ensure the graduation of competent endoscopists in an environment of evolving resident training guidelines.
